# Exploratory Analysis of Bone Marrow IL-10 and Plasma IL-1RA in Acute Traumatic Versus Chronic Non-Traumatic Orthopedic Patients: A Prospective Observational Study

**DOI:** 10.3390/pathophysiology33030071

**Published:** 2026-09-21

**Authors:** Muriel Tahtouh Zaatar, Alex Kattoura, Bayan Aasar, Rima Othman, Souheil Hallit, Marc Karam, Ziad Fajloun, Rabih Roufayel, Ramy Haroun, Rita Khalil, Boutros Tannoury, Francis Kattoura, Majd El Hajj Moussa, Jean-Claude Lahoud, Charbel Tawk, Fadi Hoyek

**Affiliations:** 1 Department of Biological and Physical Sciences, American University in Dubai, Dubai P.O. Box 28282, United Arab Emirates; 2 School of Medicine and Medical Sciences, Holy Spirit University of Kaslik (USEK), Jounieh P.O. Box 446, Lebanon; souheilhallit@usek.edu.lb (S.H.); ramy.r.haroun@net.usek.edu.lb (R.H.); rita.ta.khalil@net.usek.edu.lb (R.K.); boutros.j.eltannoury@net.usek.edu.lb (B.T.); majd.g.elhajjmoussa@net.usek.edu.lb (M.E.H.M.); jeanclaudelahoud@usek.edu.lb (J.-C.L.); charbelmtawk@usek.edu.lb (C.T.); fadihoyek@usek.edu.lb (F.H.); 3 Faculty of Sciences, University of Balamand, Al-Kourah, Tripoli P.O. Box 100, Lebanon; bayan.aasar@std.balamand.edu.lb (B.A.); marc.karam@balamand.edu.lb (M.K.); 4 Department of Pulmonary Medicine and Critical Care, Johns Hopkins University, Baltimore, MD 21205, USA; remaothman3@gmail.com; 5 Department of Psychology, College of Humanities, Effat University, Jeddah 21478, Saudi Arabia; 6 Applied Science Research Center, Applied Science Private University, P.O. Box 166, Amman 11931, Jordan; 7 Department of Cell Culture, Laboratory of Applied Biotechnology (LBA3B), Azm Center for Research in Biotechnology and Its Applications, EDST, Lebanese University, Tripoli P.O. Box 100, Lebanon; ziad.fajloun@ul.edu.lb; 8 Faculty of Sciences 3, Lebanese University, Michel Slayman Tripoli Campus, Ras Maska 1352, Lebanon; 9 College of Engineering and Technology, American University of the Middle East, Egaila 54200, Kuwait; rabih.roufayel@aum.edu.kw; 10 Department of Orthopedic Surgery, Notre Dame de Secours University Hospital Center, Byblos P.O. Box 3, Lebanon; 11 Aspetar Orthopaedic and Sports Medicine Hospital, Doha P.O. Box 29222, Qatar; fkattoura@aspetar.com

**Keywords:** cytokines, inflammation, traumatic injury, non-traumatic injury, orthopedic surgery, bone marrow, plasma, ELISA, interleukin

## Abstract

**Introduction**: Inflammation is a central component of the biological processes involved in orthopedic interventions, whether performed for acute traumatic injuries or chronic non-traumatic conditions. Cytokines play major roles in modulating these responses; however, data describing cytokine activity in bone marrow, particularly during orthopedic procedures, remain limited. This study aimed to examine variations in cytokine levels in traumatic and non-traumatic orthopedic surgeries by analyzing simultaneous bone marrow and plasma samples. **Methods**: This single-center prospective observational study with exploratory objectives included skeletally mature patients undergoing urgent surgery for acute traumatic fractures or planned surgery for chronic non-traumatic degenerative orthopedic conditions. Bone-marrow and peripheral plasma samples were collected simultaneously intraoperatively. All samples were tested in duplicate for interleukin-8 (IL-8), interleukin-6 (IL-6), interleukin-10 (IL-10), interleukin-4 (IL-4), interleukin-1 receptor antagonist (IL-1RA), and tumor necrosis factor-α (TNF-α) using developmental ELISA kits. Cytokine levels were compared between traumatic and non-traumatic groups and between bone-marrow and plasma samples. **Results**: Nineteen participants were included, providing 19 bone-marrow samples and 15 plasma samples, with the number of available measurements varying among cytokines. Bone-marrow IL-10 was detected in three of five available traumatic samples and in none of the eight available non-traumatic samples (unadjusted *p* = 0.035; false-discovery-rate-adjusted *q* = 0.178). Plasma IL-1RA concentrations were higher in traumatic than in non-traumatic patients (median 2828.00 versus 1269.00 pg/mL; unadjusted *p* = 0.038; *q* = 0.178). Bone-marrow IL-8 also showed higher concentrations in traumatic patients and approached the unadjusted significance threshold (*p* = 0.053; *q* = 0.178). No comparison remained statistically significant after FDR correction. **Discussion**: Bone-marrow IL-10 detection and plasma IL-1RA concentrations showed nominal between-group differences that did not remain statistically significant after multiple-comparison adjustment. Although preliminary and hypothesis-generating, these observations identify potentially informative local and systemic cytokine patterns and highlight the value of further investigating bone-marrow cytokine responses in orthopedic trauma.

## 1. Introduction

Inflammation is a central component of the biological processes involved in orthopedic interventions, whether these procedures address acute traumatic injuries or chronic elective conditions. Urgent surgeries, typically required following acute trauma, are generally associated with greater tissue damage and systemic stress. Consequently, they elicit more pronounced inflammatory responses compared with elective procedures [1].

Cytokines play a fundamental role in coordinating inflammatory cascades and modulating immune responses, tissue repair, and systemic reactions triggered by injury [2]. Dysregulation of cytokine activity can significantly influence patient outcomes, contributing to complications such as systemic inflammatory response syndrome (SIRS), multi-organ dysfunction syndrome (MODS), and delayed recovery [2,3,4].

Among the cytokines involved in trauma-related inflammation, interleukin-6 (IL-6), interleukin-8 (IL-8), and tumor necrosis factor-α (TNF-α) are among the most extensively studied. These mediators are rapidly released following tissue injury and hemorrhage, promoting both local and systemic inflammatory responses [5]. Elevated IL-6 and IL-8 levels have been associated with trauma severity, length of hospital stay, and increased risk of complications such as acute respiratory distress syndrome (ARDS) and MODS [6]. However, findings regarding the relationship between cytokine levels and clinical manifestations, including pain intensity, remain inconsistent [2].

Beyond their systemic effects, cytokines within the bone marrow contribute significantly to the regulation of local inflammatory responses. This local activity influences bone regeneration and may affect the development of post-traumatic complications, including osteoarthritis [7]. In elective orthopedic procedures, which are typically performed under controlled and minimally traumatic conditions, cytokine activation tends to be more localized and regulated, although it remains relevant to healing and tissue repair [8].

Monitoring cytokine responses is therefore critical for optimizing perioperative outcomes. Dysregulated cytokine activity may contribute to prolonged pain, delayed fracture healing, and post-traumatic osteoarthritis, particularly in trauma patients with heightened inflammatory responses [1,7]. These patients also face an increased risk of sepsis and long-term functional impairment, highlighting the importance of personalized perioperative management strategies.

Morley et al. emphasized the value of examining cytokine activity within the bone marrow, noting that bone marrow-derived cytokine profiles may provide deeper insight into inflammatory dynamics during orthopedic procedures [3]. As an important immunological microenvironment, the bone marrow may better reflect localized inflammatory processes than peripheral blood; however, it remains relatively understudied in this context. Most existing investigations focus primarily on systemic cytokine levels, leaving a substantial knowledge gap regarding cytokine behavior within the bone marrow compartment.

## 2. Problem Statement

Inflammation represents both a necessary reparative process and a potential source of complications in orthopedic surgery. While systemic cytokine profiles have been widely investigated, the role of bone marrow-derived cytokines remains insufficiently explored. Orthopedic procedures performed for acute traumatic injuries are associated with intense inflammatory responses that may lead to SIRS, MODS, and post-traumatic osteoarthritis, whereas elective surgeries are performed under more controlled conditions and generate distinct inflammatory patterns. Limited data regarding bone-marrow cytokine variability in traumatic and non-traumatic orthopedic settings restrict current understanding of local and systemic inflammatory patterns.

Accordingly, this exploratory observational study aimed to compare intraoperative bone-marrow and plasma cytokine measurements between patients undergoing surgery for acute traumatic fractures and those undergoing surgery for chronic non-traumatic degenerative orthopedic conditions, and to explore differences between the two biological compartments.

## 3. Methodology

Patients who had reached skeletal maturity and were undergoing surgical treatment for degenerative disease or fractures involving long-bone diaphysis or hip and knee articulations were invited to participate. The study received approval from the local ethics committee, and written informed consent was obtained from all participants or their legal guardians prior to surgery. Exclusion criteria included pregnancy, malignancy, immunodeficiency, bone marrow abnormalities, and autoimmune or rheumatic diseases. The study was conducted over a 9-month period from March 2024 to December 2024. Patient confidentiality was maintained throughout the study, and demographic and clinical variables, including sex, age, cause of injury, type of intervention, and cytokine levels, were recorded.

This study was designed as a single-center prospective observational study with exploratory objectives. The study population comprised skeletally mature patients undergoing orthopedic surgery. The comparison groups were defined according to surgical indication: the traumatic group included patients undergoing urgent operative treatment for acute fractures involving the long-bone diaphysis or hip/knee articulations, whereas the non-traumatic group included patients undergoing planned surgery for chronic degenerative orthopedic conditions. The outcomes were intraoperative bone-marrow and plasma cytokine measurements, assessed between the two clinical groups and between biological compartments. No therapeutic intervention was assigned. Several anatomical sites and operative indications were represented, and detailed fracture classification was not consistently available; therefore, both groups were clinically heterogeneous.

Before surgery, all patients were clinically and hemodynamically stabilized according to standard preoperative protocols. Bone marrow was collected intraoperatively from the operative long-bone or hip/knee surgical site before the reparative stage of the procedure. Peripheral venous blood was collected at the same operative time into potassium ethylenediaminetetraacetic acid tubes. The precise anatomical marrow-collection location and collected volume were not documented consistently for every participant and could not be reliably reconstructed retrospectively. Both bone-marrow and plasma samples were centrifuged immediately at 1500 rpm for 10 min using the same laboratory centrifuge configuration and were subsequently stored at −72 °C until analysis. Each sample was tested in duplicate for interleukin-8 (IL-8), interleukin-6 (IL-6), interleukin-10 (IL-10), interleukin-4 (IL-4), interleukin-1 receptor antagonist (IL-1RA), and tumor necrosis factor-α (TNF-α) using PeproTech developmental enzyme-linked immunosorbent assay kits. ELISA plates were read at an optical density of 405 nm using a microplate reader, and cytokine concentrations were expressed in pg/mL. Samples requiring twofold or threefold dilution were corrected accordingly during data processing. Nineteen bone-marrow samples and 15 plasma samples were available for analysis. A specific reason for each of the four unavailable plasma samples was not documented in the archived study records. Missing measurements were not imputed, and analyses were performed using the available observations for each cytokine. Paired bone-marrow and plasma analyses included only participants with both measurements available for the cytokine under evaluation.

Values recorded in the source dataset as “not detected” were considered to be below the assay detection threshold and were not interpreted as evidence of complete cytokine absence. Because verified assay-specific numerical limits of detection were not available for the present reanalysis, no numerical substitution based on the detection limit was performed. For rank-based analyses, non-detected measurements were assigned the lowest tied rank below detected concentrations. Blank entries were treated as missing measurements and were excluded on an available-case basis. The number of available observations is therefore reported separately for each cytokine and comparison. Complete archived information regarding assay catalog and lot numbers, assay-specific numerical limits of detection, and manufacturer-reported intra-assay and inter-assay coefficients of variation were unavailable. TNF-α was excluded from inferential analysis because the assay produced unreliable diffuse or negative readings. Cytokine results were analyzed according to sample source (bone marrow versus plasma) and patient category (traumatic versus non-traumatic). Samples that underwent dilution were mathematically adjusted to reflect their original concentrations.

## 4. Statistical Analysis

Continuous variables were summarized using medians and interquartile ranges, in accordance with the distributional characteristics of the data. Cytokine concentrations were compared between traumatic and non-traumatic patients using two-sided exact Mann–Whitney U tests. Bone-marrow and plasma concentrations were compared using two-sided exact Wilcoxon signed-rank tests, including patients with available paired measurements for the cytokine under evaluation.

For bone-marrow IL-10, all available measurements in the non-traumatic group were below the assay detection threshold. Therefore, in addition to the concentration-based comparison, the proportions of detected and non-detected samples were compared using Fisher’s exact test. TNF-α was not included in the inferential analyses because the corresponding assay results did not meet the required analytical quality criteria.

To account for the evaluation of multiple cytokines, the Benjamini–Hochberg false-discovery-rate procedure was applied separately to the between-group analyses and the paired bone-marrow-versus-plasma analyses. Both unadjusted *p*-values and false-discovery-rate-adjusted q-values are reported. A two-sided *p*-value below 0.05 indicated an unadjusted statistical difference, while a q-value below 0.05 indicated statistical significance after adjustment for multiple comparisons.

Effect sizes were estimated using Cliff’s delta for independent-group comparisons and matched rank-biserial correlations for paired comparisons, with 95% confidence intervals when estimable. Statistical analyses were performed using Python version 3.13, SciPy version 1.17.0, and statsmodels version 0.14.6.

No a priori sample-size calculation was performed because recruitment was limited to the predefined nine-month study period. The analyses should therefore be interpreted as exploratory, and nonsignificant comparisons should not be considered evidence of equivalence or absence of biological differences.

## 5. Results

A total of 19 participants were enrolled in the study, providing 19 bone-marrow samples (nine traumatic and 10 non-traumatic) and 15 plasma samples (six traumatic and nine non-traumatic). The mean age of the cohort was 65.79 ± 16.99 years, and 52.6% of participants were male. Baseline demographic and cytokine descriptive statistics are presented in Table 1. 

### 5.1. Differences in Cytokine Concentrations Between Traumatic and Non-Traumatic Patients

Cytokine distributions and the number of available measurements varied among the analytes; therefore, the results are presented as medians and interquartile ranges.

Bone-marrow IL-10 was detected in three of five available traumatic samples and in none of the eight available non-traumatic samples. The difference in detection frequency met the unadjusted significance threshold using Fisher’s exact test (*p* = 0.035), although it did not remain statistically significant after false-discovery-rate adjustment (*q* = 0.178). The median bone-marrow IL-10 concentration was 83.50 pg/mL [IQR 0.00–200.25] in traumatic patients and 0.00 pg/mL [IQR 0.00–0.00] in non-traumatic patients.

Plasma IL-1RA concentrations were higher in traumatic patients, with a median of 2828.00 pg/mL [IQR 2229.00–3223.75], compared with 1269.00 pg/mL [IQR 1144.00–1499.00] in non-traumatic patients. This difference met the unadjusted significance threshold using the exact Mann–Whitney U test (*p* = 0.038), but not after false-discovery-rate adjustment (*q* = 0.178).

Bone-marrow IL-8 concentrations were also higher in traumatic patients, with a median of 263.30 pg/mL [IQR 188.62–470.92], compared with 146.65 pg/mL [IQR 85.80–233.87] in non-traumatic patients. This comparison approached, but did not reach, the unadjusted significance threshold (*p* = 0.053; *q* = 0.178).

**Figure 1:**
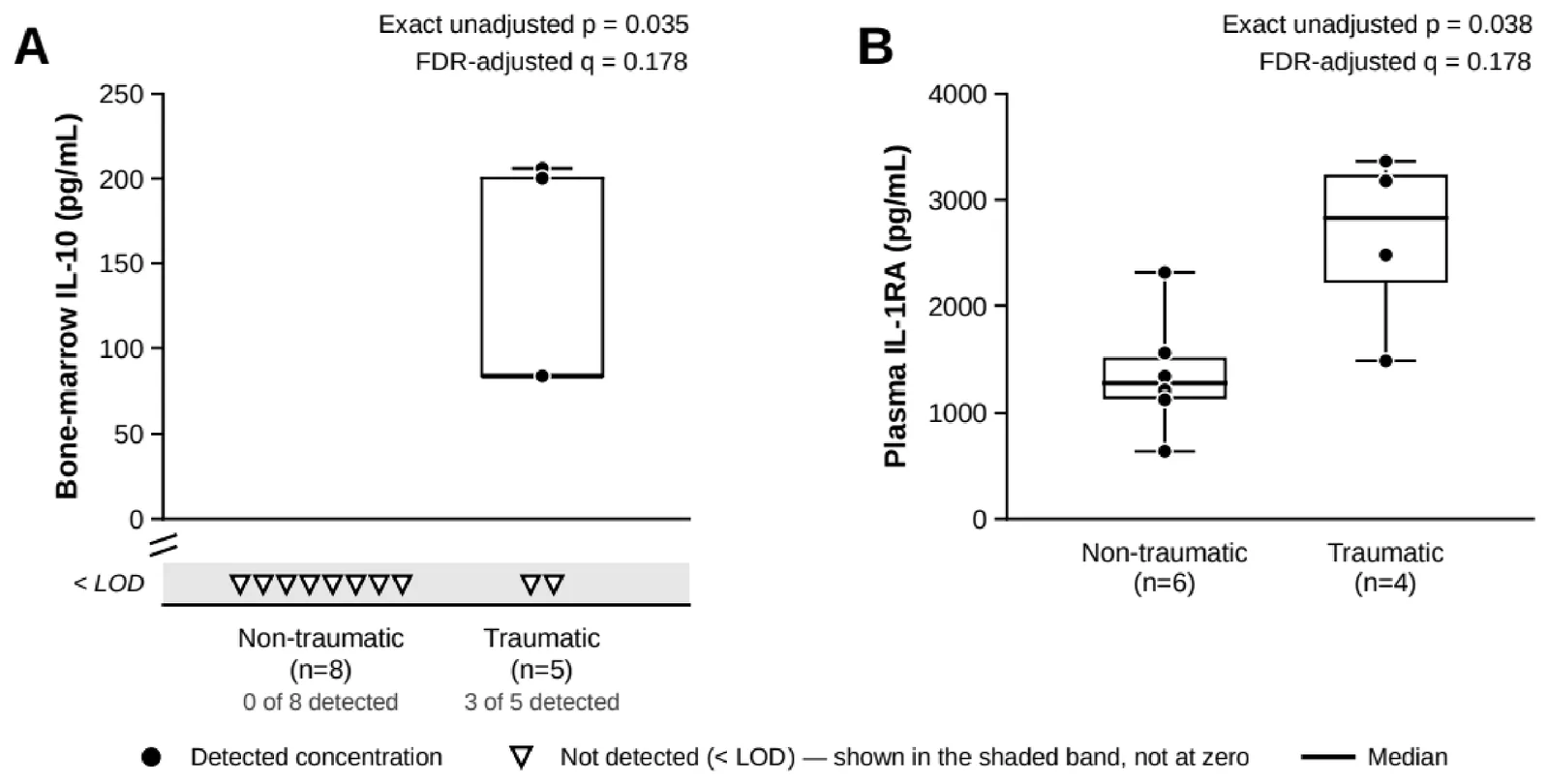
**Bone-marrow IL-10 and plasma IL-1RA measurements in traumatic and non-traumatic orthopedic patients.** (**A**) Bone-marrow IL-10 measurements. Detected concentrations are shown as filled circles at their measured values. Measurements recorded as “not detected” are shown separately as open inverted triangles in the shaded <LOD band and are not presented as measured concentrations of zero. Each open inverted triangle represents one non-detected sample. For Panel A, the box-and-whisker summary is based on detected concentrations only. Missing measurements are not plotted. The unadjusted *p*-value was calculated using Fisher’s exact test comparing detected and non-detected samples. (**B**) Plasma IL-1RA concentrations. Individual points represent available patient measurements; boxes indicate the interquartile range, horizontal lines indicate the median, and whiskers represent the observed range. Groups were compared using the two-sided exact Mann–Whitney U test. False-discovery-rate-adjusted q-values were calculated using the Benjamini–Hochberg procedure across the 10 retained between-group comparisons.

No other between-group comparison met the unadjusted or adjusted significance thresholds. TNF-α was excluded from inferential analysis because the assay results did not meet the required analytical quality criteria. Complete comparisons are presented in Table 2, and the principal exploratory findings are illustrated in Figure 1.

### 5.2. Paired Bone-Marrow and Plasma Comparisons

Paired analyses included only participants with both bone-marrow and plasma measurements available for the cytokine under evaluation. In the traumatic group, no bone-marrow-versus-plasma comparison reached statistical significance after false-discovery-rate adjustment (Table 3). In the non-traumatic group, no corresponding paired comparison reached statistical significance after false-discovery-rate adjustment (Table 4). Several cytokines showed differences in median concentrations between compartments; however, the limited number of complete pairs did not allow firm conclusions regarding compartment-specific cytokine patterns. A correlation between bone-marrow IL-10 and plasma IL-1RA was not estimated because only two traumatic participants had complete measurements for both variables, which was insufficient for a meaningful correlation analysis.

Figure 1 illustrates the two principal exploratory findings from the between-group analyses. Figure 1A shows the distribution of bone-marrow IL-10 measurements in traumatic and non-traumatic patients, and Figure 1B shows plasma IL-1RA concentrations in the two groups. Both comparisons met the unadjusted significance threshold but did not remain statistically significant after false-discovery-rate adjustment.

## 6. Discussion

Bone-marrow IL-8 concentrations tended to be higher in traumatic patients, whereas IL-6 did not show a clear between-group difference in either compartment (Table 2). However, these increases did not reach statistical significance for IL-6 in either plasma (*p* = 0.929) or bone marrow (*p* = 0.329). These trends are consistent with previous studies, such as that of Volpin G, who reported elevated IL-6 and IL-8 levels following traumatic injuries and emphasized their role as key mediators of acute inflammation [1]. The lack of statistical significance in our cohort may reflect several factors, including injury severity, limited sample size, timing of sample collection, and individual immunological variability. Additionally, Zhou D demonstrated that IL-6 may also increase in response to non-traumatic stressors, including psychological and physical stress, suggesting that elevated IL-6 is not exclusive to trauma [9].

Bone-marrow IL-8 concentrations approached the unadjusted significance threshold (*p* = 0.053) following traumatic injury compared with chronic non-traumatic conditions. This finding may relate to IL-8’s established role in local inflammatory signaling and its association with receptor activator of nuclear factor kappa-B ligand (RANKL)-mediated osteoclastic activity and bone turnover [10]. Additionally, IL-8 is known to be rapidly mobilized from marrow-resident stromal cells and endothelial compartments following mechanical injury, which may explain why the earliest and most pronounced IL-8 changes appear within the bone marrow rather than systemic circulation.

Bone-marrow IL-10 and plasma IL-1RA showed potentially relevant exploratory differences between traumatic and non-traumatic patients. Bone-marrow IL-10 was detected more frequently in traumatic patients, whereas all available non-traumatic measurements were below the assay detection threshold. This pattern is broadly consistent with the report by Patilas et al., who described lower IL-10 concentrations during the chronic phase of spinal cord injury compared with the acute response [11]. Plasma IL-1RA concentrations were also higher in traumatic patients, in line with previous evidence linking IL-1RA and IL-10 within inflammatory joint environments [12]. Experimental studies have further shown that IL-10 may promote IL-1RA production in circulating immune cells [13,14]. Although the unadjusted IL-10 and IL-1RA comparisons met the conventional significance threshold, neither remained statistically significant after false-discovery-rate adjustment. These observations may identify candidate signals for further study of early post-traumatic immunoregulation; however, they do not establish a trauma-specific cytokine response or a mechanistic relationship between bone-marrow IL-10 and plasma IL-1RA. The present dataset does not permit firm characterization of a distinct inflammatory profile in chronic non-traumatic conditions. Chronic inflammatory diseases are generally characterized by low-grade systemic inflammation rather than the abrupt cytokine surges typically associated with acute trauma [2,13]. This distinction may contribute to the differing cytokine patterns observed between traumatic and non-traumatic conditions. Degenerative bone and joint disease is frequently characterized by paracrine rather than endocrine inflammatory signaling, which may explain the predominance of plasma rather than marrow cytokine elevations in chronic conditions.

**Figure 2:**
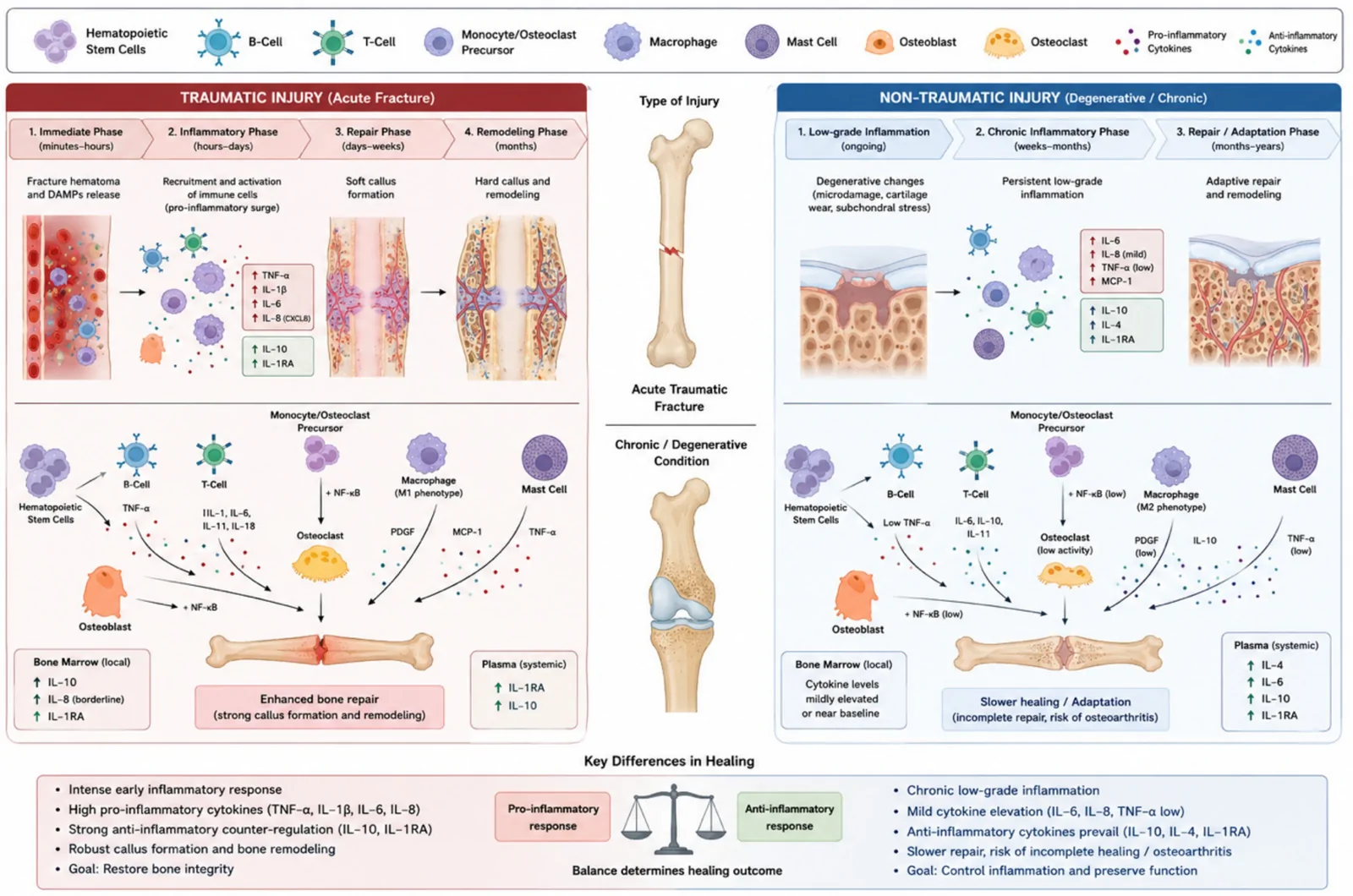
**Conceptual schematic of inflammatory and reparative responses in traumatic versus non-traumatic orthopedic injury.** The schematic summarizes general patterns of inflammatory signaling, immune-cell activity, cytokine responses, tissue repair, and remodeling in acute traumatic injury and chronic degenerative conditions. It is provided for biological context and does not represent a mechanistic model directly established by the present exploratory study. Red and green upward arrows denote increases in pro-inflammatory and anti-inflammatory cytokines, respectively; these arrows are conceptual and not quantitative.

To provide broader biological context, Figure 2 presents a conceptual overview of inflammatory and reparative responses in acute traumatic and chronic non-traumatic orthopedic conditions. The illustration summarizes general patterns of inflammatory timing, immune-cell activity, cytokine signaling, tissue repair, and remodeling. It is included for contextual purposes and should not be interpreted as a mechanistic model derived directly from the current dataset. 

Our nonsignificant findings for bone-marrow IL-6 (*p* = 0.329) can be considered alongside observations by Morley JR, who reported higher IL-6 concentrations in bone marrow than in peripheral blood [3]. However, the present data do not establish preferential marrow cytokine production or the direction, timing, or mechanisms of compartment-specific inflammatory responses. 

Paired comparisons between bone-marrow and plasma concentrations did not identify statistically significant compartmental differences in either the traumatic or non-traumatic group after false-discovery-rate adjustment (Table 3 and Table 4). Although some cytokines showed differences in median concentrations between compartments, the number of complete pairs was small and varied substantially among analytes. Previous work has described higher bone-marrow IL-6 concentrations than peripheral blood concentrations, supporting continued investigation of local inflammatory activity within the marrow compartment [3]. IL-10 has also been reported to promote IL-1RA production in circulating immune cells, providing biological context for considering possible relationships between anti-inflammatory cytokine pathways [14]. However, the present findings do not permit firm conclusions regarding compartment-specific cytokine regulation or a direct mechanistic link between bone-marrow IL-10 and plasma IL-1RA. The conceptual framework shown in Figure 2, together with the previous literature [15,16], provides broader context for the hypothesis that traumatic injury may involve coordinated local and systemic inflammatory responses.

From a clinical perspective, the present findings do not support the use of bone-marrow IL-10 or plasma IL-1RA as diagnostic, prognostic, or therapeutic biomarkers and should not alter current perioperative management. The principal clinical relevance of this exploratory observational study is methodological and hypothesis-generating: simultaneous intraoperative bone-marrow and plasma sampling allowed direct comparison of local and systemic cytokine measurements in an underexplored setting. Whether marrow cytokine profiling provides clinically useful information beyond peripheral blood cannot be determined from this sample. This study has several limitations. The cohort was small and clinically heterogeneous, and the number of available measurements differed among cytokines. The study was conducted at a single center and included only one intraoperative sampling time point. The limited sample size did not permit reliable adjustment for potential confounding factors such as age, sex, fracture type, anatomical site, injury severity, timing from trauma to surgery, comorbidities, and preoperative medication exposure. Sampling location and operative indication may also have influenced local cytokine concentrations. Accordingly, the findings should be considered exploratory and hypothesis-generating. In addition, the exact anatomical marrow-collection site and collected volume were not recorded consistently for every participant, and the reasons for individual unavailable plasma samples could not be reconstructed from the archived records. Complete assay-specific limits of detection and manufacturer-reported intra-assay and inter-assay coefficients of variation were also unavailable. These limitations restrict the assessment of analytical reproducibility and may have contributed to variability in the cytokine measurements. Larger prospective studies with more homogeneous populations, standardized sampling sites, complete paired measurements, and predefined clinical covariates are needed to confirm the observed patterns. 

## 7. Conclusions

This prospective observational study identified nominally higher bone-marrow IL-10 detection and higher plasma IL-1RA concentrations in traumatic orthopedic patients compared with non-traumatic patients. Neither finding remained statistically significant after false-discovery-rate adjustment, and these observations therefore do not establish trauma-specific cytokine regulation or support immediate clinical application. Simultaneous intraoperative collection of bone-marrow and plasma samples enabled exploratory assessment of local and systemic cytokine measurements within the same study framework.

## Figures and Tables

**Table 1:** **Sociodemographic characteristics and overall cytokine concentrations of the study sample (N = 19).** Cytokine concentrations are presented as median [interquartile range] because of their skewed distributions and the presence of non-detected measurements. The number of available observations is reported separately for each cytokine. Values recorded as “not detected” are displayed as 0.00 for descriptive presentation and were assigned the lowest tied rank in rank-based analyses. TNF-α was excluded because the assay produced unreliable diffuse or negative readings. Traumatic cases comprised acute fractures involving the long-bone diaphysis or hip/knee articulations, whereas non-traumatic cases comprised chronic degenerative orthopedic conditions. Injury Severity Score, participant-level AO/OTA fracture classification, exact trauma-to-surgery interval, and exact anatomical marrow-collection site were not consistently available in the archived records and therefore could not be summarized reliably.

**Variable**	**Total Sample (N = 19)**	**Traumatic (n = 9)**	**Non-Traumatic (n = 10)**
Age, years, mean ± SD	65.79 ± 16.99	60.00 ± 22.02	71.00 ± 9.08
**Sex, n (%)**			
Male	10 (52.6%)	6 (66.7%)	4 (40.0%)
Female	9 (47.4%)	3 (33.3%)	6 (60.0%)
**Variable**	**Overall median [IQR]**		
**Bone-marrow cytokines, pg/mL**			
IL-8, n = 19	194.00 [104.50–321.00]		
IL-10, n = 13	0.00 [0.00–0.00]		
IL-6, n = 16	7.18 [0.00–48.61]		
IL-4, n = 6	0.00 [0.00–20.69]		
IL-1RA, n = 10	931.00 [488.25–2681.25]		
**Plasma cytokines, pg/mL**			
IL-8, n = 15	135.08 [127.29–152.77]		
IL-10, n = 13	25.50 [0.00–60.50]		
IL-6, n = 13	37.91 [0.00–52.91]		
IL-4, n = 6	63.32 [47.07–92.37]		
IL-1RA, n = 10	1516.50 [1236.50–2437.75]		

**Table 2:** Cytokine concentrations in traumatic and non-traumatic orthopedic patients.

Compartment and Cytokine	Traumatic, n	Traumatic Median [IQR], pg/mL	Non-Traumatic, n	Non-Traumatic Median [IQR], pg/mL	Unadjusted *p*-Value	FDR-Adjusted q-Value	Cliff’s Delta [95% CI]
**Bone marrow**							
IL-8	9	263.30 [188.62–470.92]	10	146.65 [85.80–233.87]	0.053	0.178	0.53 [0.04 to 0.91]
IL-10	5	83.50 [0.00–200.25]	8	0.00 [0.00–0.00]	0.035 *	0.178	0.60 [0.20 to 1.00]
IL-6	9	14.18 [0.00–134.00]	7	0.00 [0.00–18.36]	0.329	0.548	0.30 [−0.25 to 0.78]
IL-4	2	0.00 [0.00–0.00]	4	13.79 [0.00–30.68]	0.467	0.583	−0.50 [−1.00 to 0.00]
IL-1RA	3	2328.00 [1573.00–7980.00]	7	549.00 [458.50–1921.50]	0.267	0.533	0.52 [−0.14 to 1.00]
**Plasma**							
IL-8	6	146.90 [128.49–234.17]	9	135.08 [127.58–145.65]	0.388	0.555	0.30 [−0.41 to 0.85]
IL-10	6	55.50 [12.62–79.62]	7	0.00 [0.00–25.50]	0.244	0.533	0.38 [−0.24 to 1.00]
IL-6	6	35.27 [6.68–98.70]	7	37.91 [5.09–49.05]	0.929	0.929	0.05 [−0.62 to 0.67]
IL-4	2	48.79 [24.40–73.19]	4	63.32 [48.99–100.16]	0.800	0.889	−0.25 [−1.00 to 1.00]
IL-1RA	4	2828.00 [2229.00–3223.75]	6	1269.00 [1144.00–1499.00]	0.038	0.178	0.83 [0.33 to 1.00]

Data are presented as median [interquartile range, IQR]. Sample sizes vary because measurements were not available for every cytokine in every participant. Except where indicated, *p*-values were calculated using two-sided exact Mann–Whitney U tests. * The bone-marrow IL-10 p-value was calculated using Fisher’s exact test comparing detected and non-detected samples because all available non-traumatic measurements were below the detection threshold. q-Values were calculated using the Benjamini–Hochberg false-discovery-rate procedure across the 10 retained between-group comparisons. Effect sizes are presented as Cliff’s delta with 95% confidence intervals. TNF-α was excluded from inferential analysis because the assay results did not meet the required analytical quality criteria. No comparison remained statistically significant after false-discovery-rate adjustment.

**Table 3:** Paired comparison of bone-marrow and plasma cytokine concentrations in traumatic patients.

Cytokine	Complete Pairs, n	Bone Marrow Median, pg/mL	Plasma Median, pg/mL	Unadjusted *p*-Value	FDR-Adjusted q-Value	Matched Rank-Biserial Effect
IL-8	6	321.57	146.9	0.219	0.643	0.62
IL-10	4	41.75	73.25	0.250	0.643	−0.80
IL-6	6	7.09	35.27	1	1.000	0
IL-4	1	0.00	0.00	Not estimable	Not estimable	Not estimable
IL-1RA	2	7225	2421.5	1	1.000	0.33

**Table 4:** Paired comparison of bone-marrow and plasma cytokine concentrations in non-traumatic patients.

Cytokine	Complete Pairs, n	Bone Marrow Median, pg/mL	Plasma Median, pg/mL	Unadjusted *p*-Value	FDR-Adjusted q-Value	Matched Rank-Biserial Effect
IL-8	9	179	135.08	0.359	0.643	0.38
IL-10	6	0	0	0.500	0.643	−1.00
IL-6	7	0	37.91	0.438	0.643	−0.43
IL-4	2	13.79	48.03	0.500	0.643	−1.00
IL-1RA	6	508.5	1269	0.219	0.643	−0.62

Data are based only on participants with both bone-marrow and plasma measurements available for the cytokine under evaluation. *p*-Values were calculated using two-sided exact Wilcoxon signed-rank tests. The traumatic IL-4 comparison was not estimable because only one complete pair was available. q-Values were calculated using the Benjamini–Hochberg false-discovery-rate procedure across the nine estimable paired comparisons. TNF-α was excluded because the assay results did not meet the required analytical quality criteria.

## Data Availability

The data supporting the findings of this study are available from the corresponding author upon reasonable request. Restrictions apply to the availability of these data, which were used under institutional license and therefore are not publicly accessible. Data may be shared with permission from the Ethics Committee.

## References

[1] Volpin G., Cohen M., Assaf M., Meir T., Katz R., Pollack S. (2014). Cytokine Levels (IL-4, IL-6, IL-8 and TGFβ) as Potential Biomarkers of Systemic Inflammatory Response in Trauma Patients. Int. Orthop..

[2] Li R., Ye J.J., Gan L., Zhang M., Sun D., Li Y., Wang T., Chang P. (2024). Traumatic inflammatory response: Pathophysiological role and clinical value of cytokines. Eur. J. Trauma Emerg. Surg..

[3] Morley J.R., Smith R.M., Pape H.C., MacDonald D.A., Trejdosiewitz L.K., Giannoudis P.V. (2008). Stimulation of the local femoral inflammatory response to fracture and intramedullary reaming: A preliminary study of the source of the second hit phenomenon. J. Bone Jt. Surg. Br..

[4] Sharma V., Bhardwaj N., Khurana S., Aggarwal R., Sharma N., Mathur P. (2019). Impact of monocytic cytokines in polytrauma patients with orthopedics injures. J. Clin. Orthop. Trauma.

[5] Haller J.M., McFadden M., Kubiak E.N., Higgins T.F. (2015). Inflammatory Cytokine Response Following Acute Tibial Plateau Fracture. J. Bone Jt. Surg..

[6] Aneja A., Landy D.C., Mittwede P.N., Albano A.Y., Teasdall R.J., Isla A., Kavolus M. (2022). Inflammatory cytokines associated with outcomes in orthopedic trauma patients independent of New Injury Severity score: A pilot prospective cohort study. J. Orthop. Res..

[7] Lieberthal J., Sambamurthy N., Scanzello C.R. (2015). Inflammation in joint injury and post-traumatic osteoarthritis. Osteoarthr. Cartil..

[8] Iversen I.J., Pham T.M., Schmal H. (2021). Do acute inflammatory cytokines affect 3- and 12-month postoperative functional outcomes–a prospective cohort study of 12 patients with proximal tibia fractures. BMC Musculoskelet. Disord..

[9] Zhou D., Kusnecov A.W., Shurin M.R., DePaoli M., Rabin B.S. (1993). Exposure to physical and psychological stressors elevates plasma interleukin 6: Relationship to the activation of hypothalamic-pituitary-adrenal axis. Endocrinology.

[10] Bendre M.S., Montague D.C., Peery T., Akel N.S., Gaddy D., Suva L.J. (2003). Interleukin-8 stimulation of osteoclastogenesis and bone resorption is a mechanism for the increased osteolysis of metastatic bone disease. Bone.

[11] Patilas C., Varsamos I., Galanis A., Vavourakis M., Zachariou D., Marougklianis V., Kolovos I., Tsalimas G., Karampinas P., Kaspiris A. (2024). The Role of Interleukin-10 in the Pathogenesis and Treatment of a Spinal Cord Injury. Diagnostics.

[12] Pham T.M., Erichsen J.L., Kowal J.M., Overgaard S., Schmal H. (2021). Elevation of Pro-Inflammatory Cytokine Levels Following Intra-Articular Fractures—A Systematic Review. Cells.

[13] Meert L., Mertens M.G., Meeus M., Vervullens S., Baert I., Beckwée D., Verdonk P., Smeets R.J. (2023). Identification of Metabolic Factors and Inflammatory Markers Predictive of Outcome after Total Knee Arthroplasty in Patients with Knee Osteoarthritis: A Systematic Review. Int. J. Environ. Res. Public Health.

[14] Cassatella M.A., Meda L., Gasperini S., Calzetti F., Bonora S. (1994). Interleukin 10 (IL-10) upregulates IL-1 receptor antagonist production from lipopolysaccharide-stimulated human polymorphonuclear leukocytes by delaying mRNA degradation. J. Exp. Med..

[15] Saed A., Neal-Smith G., Fernquest S., Bourget-Murray J., Wood A. (2023). Management of complex regional pain syndrome in trauma and orthopaedic surgery: A systematic review. Br. Med. Bull..

[16] Lagas I.F., Nijst B.K., Hartgens F., de Bie R.A., Vis M., Verhaar J.A., de Vos R.J. (2024). Lower extremity tendinopathies are associated with metabolic and chronic diseases: A systematic review. Muscles Ligaments Tendons J..

